# Embedding Child Rights Principles and Practises in Mega Sport Event Planning

**DOI:** 10.3389/fspor.2021.695666

**Published:** 2021-09-10

**Authors:** Oluwaseyi Aina, David McGillivray, Sandro Carnicelli, Gayle McPherson

**Affiliations:** Centre for Culture, Sport and Events, University of the West of Scotland, Paisley, United Kingdom

**Keywords:** Olympic Games, human rights, child rights, mega sporting events, Tokyo 2020 Olympics

## Abstract

Recently, there has been growing concern about the lack of intentionality of mega sport event (MSE) organisers in ensuring that child rights are adequately respected, protected and promoted before, during, and after the events take place. In the context of the summer Olympic Games, reported child rights infringements have been on the rise, both in relation to abuse in sport itself and the negative consequences associated with planning and delivering the Games. In response to reports of child rights infringements, a coalition of actors, including non-governmental and civil society organisations have sought to pressure event owners and organisers to strengthen protections in the planning and delivery of their events. To date, however, child rights commitments have not been fully embedded in policies and principles guiding the planning and delivery of the Olympic Games. In this article, we explore the field of child rights in the context of the Olympic Games, focusing on a case study of the Tokyo 2020 edition. Drawing on documentary analysis and semi-structured interviews with Tokyo 2020 stakeholders and affiliates, detailed appraisal of the planning process was undertaken. Findings show that while the Japanese authorities have signed up to international child rights conventions and embedded some child participation strategies in Games-related activity, there was little evidence that Tokyo 2020 organisers had developed or implemented robust policies, principles or practises to respect, protect and promote child rights in Games planning. This absence, we argue, is because there was no requirement to embed child rights commitments during the bidding or planning phases, as the IOC had yet to enshrine human rights in its host city contract when the Games were first awarded to Tokyo. In conclusion, we argue that it is imperative the IOC embeds child rights principles and protocols in the bidding and planning processes to ensure that the risks to children are foregrounded and acted upon by host cities and their partners, elevating human rights to a position equal to other Games requirements. This study is of international significance as the evidence will aid future host city bidders to ensure children's rights are embedded in MSE policies for each nation.

## Introduction

The term human rights gained wider currency in the middle of the 20th century (Griffin, 2008). Since its introduction, various social, economic, political and ideological struggles have been played out in the name of human rights (Horne, 2017). Debates about the role of human rights, how they are infringed and the role of international conventions in upholding rights, have become significant to discussions about free and fair global societies. Human rights have received significant sociological attention (Levy and Sznaider, 2006; Hynes et al., 2010) but more recently the terrain of sport has been at the forefront of debates about the changing dynamics of the human rights concept, especially in the context of mega sport events (MSEs) (Kidd and Donnelly, 2000; Talbot and Carter, 2018; McGillivray et al., 2019, 2021).

Since the early 2000s, the human rights agenda has become heavily contested in the context of MSEs as the International Olympic Committee (IOC) awarded the rights to host the 2008 Olympic Games to Beijing, China. Awarding the Olympic Games to Beijing despite China's poor human rights record generated intensive critique of the IOC and its partners from the international community. This critique was led by human rights non-governmental organisations (NGOs) including Human Rights Watch and Amnesty International who criticised the IOC for failing to uphold its own Olympic Charter and the values contained within it (Adi and Miah, 2011). Kidd (2010) has argued that the IOC appeared complicit in the Chinese Government's crackdown on open protest and public dissent in the lead up to the hosting of the 2008 Games. As a result, the IOC suffered a serious blow to its moral authority and legitimacy as a humanitarian organisation (Kidd, 2010). Since 2008, there has been change in the human rights and MSE domain. Some progress is evident, precipitated by awarding bodies facing increasing pressure from NGOs and civil society organisations (CSOs) to accept responsibility for the impact of their events on the most vulnerable segments of the population in host communities (McGillivray et al., 2021). This pressure has been amplified with growing media coverage of the human rights issue, given impetus by the emergence of independent coalition organisations including the Sport and Rights Alliance (SRA) and its successor, the Centre for Sports and Human Rights. These organisations brought NGOs, CSOs, awarding bodies, sporting federations, governments, sponsors, and labour unions together around the same table to address human rights issues. Coordinated activities by these organisations has increased pressure on the IOC, FIFA, and the Commonwealth Games Federation to develop principles, policies, and protocols to more effectively, respect, protect and promote human rights (McGillivray et al., 2019).

Research evidence suggests that staging MSEs affects some human rights more than others. In recent years the focus has often been on forced evictions and displacement of populations (Jones, 2001), violation of labour rights (Cotton and Weldon, 2014), restrictions on freedom of opinion, expression and movement (Killeen and Hertogen, 2014), direct political repression (Cottrell and Nelson, 2011), and human trafficking (including sex trafficking) (Matheson and Finkel, 2012). These human rights infringements are important and need to be addressed, but there are also other issues that are less visible in the media, or in academic debates, that are also worth further investigation, including child rights. The United Nations Convention on the Rights of the Child (UNCRC), which was drafted in 1989, outlines child rights protection measures that should be implemented by all signatories. However, like other rights conventions, there is inconsistency in its implementation and the general commitments made by signatories are rarely specific enough to address all eventualities. So, while the UNCRC states that host nations should consider the rights of the child when bidding, planning for, and delivering their MSEs, evidence suggests that insufficient consideration is given to child rights in policies guiding the Games (Caudwell and McGee, 2018; Dowse et al., 2018). In practise, despite long-standing international agreements on the importance of child rights, child protection measures have not until recently been a criterion for either bidding or social legacy planning for most MSEs, often rendering children invisible in this process (Brackenridge et al., 2015).

This study addresses a gap in respect of child rights and MSEs, taking as its focus the extent to which child rights considerations are effectively embedded in the bidding and planning stages of the Tokyo 2020 summer Olympic Games. The guiding research question is: To what extent are child rights principles and protocols embedded in the planning for the Tokyo 2020 Games? The paper begins by outlining the key literature on human rights challenges in MSEs, before focusing on the context of child rights as a specific dimension of human rights. It then considers the extent of child rights infringements that exist in relation to the planning and delivery of these events. Two major data collection methods were employed. Firstly, a systematic documentary analysis relating to the bidding and planning of the Tokyo 2020 Games was undertaken, drawing on strategies and policies relating to human rights, and child rights in particular. Secondly, interviews were conducted with seven key informants chosen based on their affiliations with, or interest in, child rights and the Tokyo 2020 Olympic Games. Findings are divided into two main themes: participation and provision for protection. These themes build on the principles of the UNCRC and Lundy's (2007) model of participation. We argue that while adopting a model of participation that includes the voices of children in the planning phase of the Olympic Games is imperative, robust protection measures must also be incorporated into the planning of MSEs if child rights are to be adequately addressed. Following discussion of the significance of the findings for the field of study, the paper concludes with a call for event owners, host cities, and policy makers to invest in bidding for, and delivering, MSEs free of the exploitation and abuse of children. We conclude with some practical recommendations as to how child rights considerations can be built into the governance arrangements for MSEs.

### Human Rights and Mega Sport Events

Horne (2017) has argued that human rights are inherently political and contingent—taking institutional, legal, and discursive forms. According to Brownell (2012), human rights are not pre-given moral truths but instead represent social constructions. A formal apparatus of human rights exists that includes the social movements that espouse and promote them, operating as part of a global civil society across borders and beyond the reach of governments (Keane, 2003). For Horne (2017) various social, economic, political and ideological contexts have been wrapped up under the moniker of human rights. Despite ongoing contestation, human rights have been enshrined in the fabric of the international community for more than half a century, since the establishment of the Universal Declaration of Human Rights (UDHR) in 1948. However, the emphasis of a human rights approach continues to shift, from its existing focus on the formal documentation of violations and abuses, towards a proactive emphasis on devising modes of protection through prevention (Caudwell and McGee, 2018).

Much of the focus of international covenants relating to respect for human rights has been concerned with avoiding violations (Orend, 2002) and yet rights continue to be abused in egregious ways around the world (Simmons, 2014). Even though human rights have long been ingrained in the legal, ethico-moral and socio-cultural fabric of nation states and the international community (Caudwell and McGee, 2018), many people in countries across the world still continue to be victims of rights infringements (Freeman, 2017), including in those nations hosting MSEs. For example, drawing on a case study of the Vancouver 2010 Winter Olympics, Matheson and Finkel (2012) detail the causal relationship between human rights infringements (specifically human trafficking) and MSE hosting.

In the past 10 years, storeys of human rights violations relating to MSEs including the Olympic Games and FIFA World Cup have been frequent (Henderson, 2016). Primarily, it is the size and scale of these events that increases the likelihood of rights infringements. The number of participants and spectators, alongside the sheer levels of organisational complexity (Malfas et al., 2004) means that vulnerable populations are subject to abuses that would have otherwise not been so acute. Human rights infringements in MSEs can be categorised into at least five areas–forced evictions, violation of labour rights, restrictions on freedom of expression, political repression and human trafficking. Forced evictions by state power were recorded ahead of the 2008 Summer Games in Beijing, with more than one million people displaced to make way for Olympic venues (Boykoff, 2016). Similar issues were experienced at the London 2012 Olympic Games, where low–income East Londoners were displaced for the construction of the Olympic stadium and related infrastructural developments (Watt, 2013). These effects were also seen in the gentrification of neighbourhoods related to Olympic developments in Rio de Janeiro (Gaffney, 2015). Beyond displacement, there have also been high profile violations of labour rights before several MSEs. Worden (2017) highlights the case of human rights of construction workers in Qatar, arguing that safeguarding and greater accountability should be a priority for FIFA. Relatedly, Akrivopoulou (2017) has documented the abuse of children in construction work for the Olympic Games. Restrictions on freedom of opinion, expression and movement have been commonplace in planning and delivery of the Olympic Games (Killeen and Hertogen, 2014; Ekberg and Strange, 2017). Commenting on the Beijing 2008 Olympic Games, Amnesty International and Human Rights Watch alleged that China had failed to keep the promises they made in 2001 when Beijing was bidding to host the Olympic Games with regards to improving their human rights record. Nations hosting MSEs have also been accused of direct political repression with Cottrell and Nelson (2011) highlighting the Mexican government's response to student anti-Olympics and anti government protests in 1968. The incidence of human trafficking has also been identified in the context of FIFA World Cups and both the summer and winter Olympic Games (Hennig et al., 2007; Matheson and Finkel, 2013; De Lisio et al., 2018). Human trafficking also includes child sexual exploitation and child labour infringements (Brackenridge et al., 2015).

However, accompanying studies that report the negative consequences of hosting MSEs on human rights is another body of literature on the progressive social objectives that can be leveraged through these events. While some commentators (e.g., Hoberman, 2008; Kidd, 2010) are critical of awarding MSEs to nations with suspect human rights records at all, another line of thought suggests that the attention and media scrutiny that accompanies MSE hosting can shine a light on hosts and event owners (such as the IOC), initiating or accelerating change (Schulenkorf and Edwards, 2012). Over recent years, influential NGOs and charities, including Amnesty International, Human Rights Watch, and UNICEF (through documents such as *Child Rights in Sports Principles)*, have actively lobbied MSE awarding bodies to enshrine human rights into every part of their operations, from vision, through bidding, into planning, delivery and legacy (MSE Platform, 2018).

Emerging from NGO and CSO lobbying, alongside the development of guidance documents for MSE organisers, is consensus on the need for awarding bodies and organisers to move away from rhetorical support in the form of paper policies and good intentions towards tangible protocols, practises and remedy measures, built into the governance arrangements for these events. However, as yet, MSEs do not have a universally agreed set of procedures that respect, protect and promote human rights and uphold their core organisational values (Henderson, 2016). To do this effectively, awarding bodies like the IOC have been urged to make the protection of human rights a condition of future host city contracts (Kidd, 2010; McGillivray et al., 2019).

### Child Rights and Mega Sport Events

**Figure 1:**
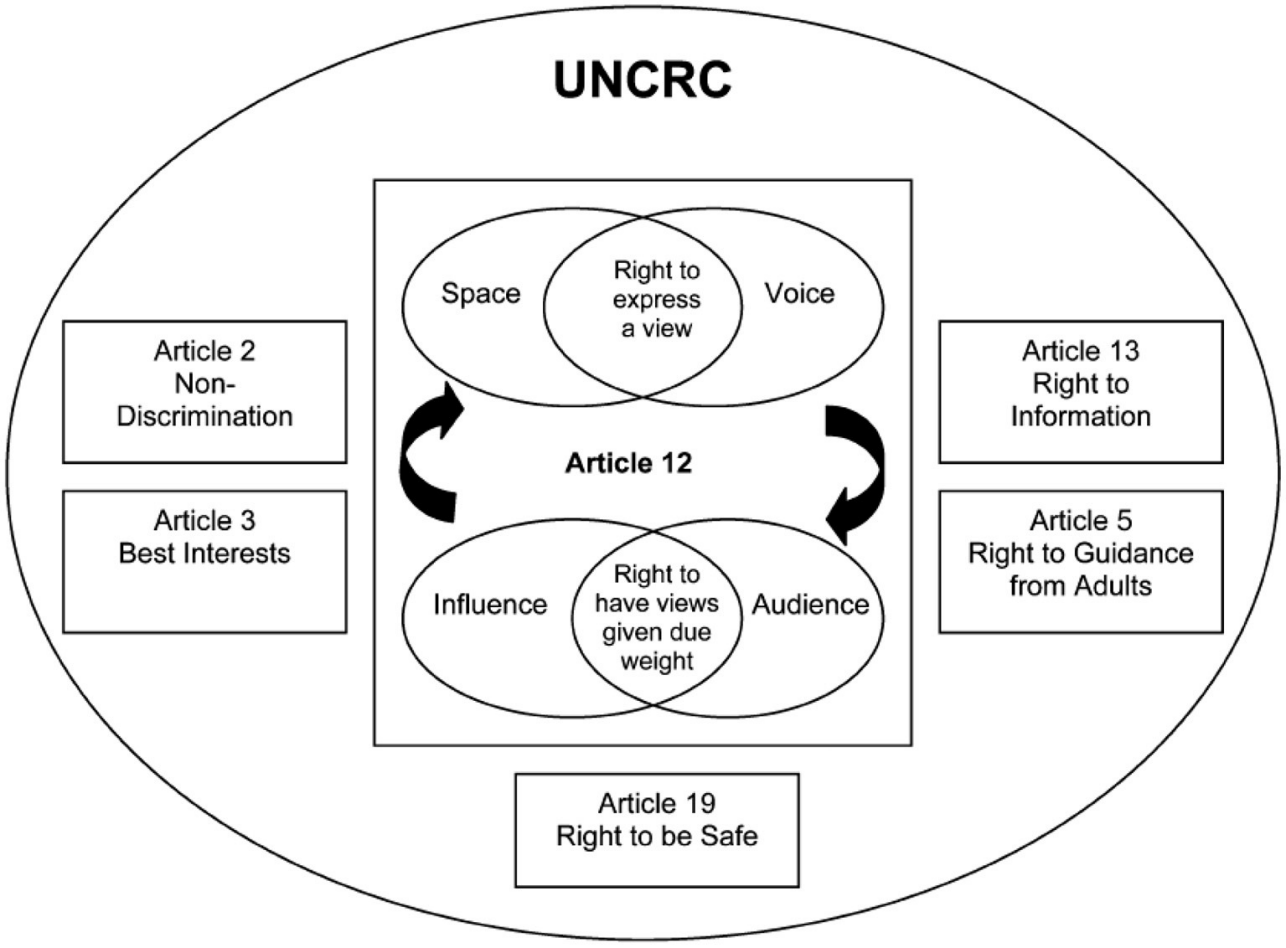
Conceptualising Article 12: Lundy (2007).

Part 1, Article 1 of the UNCRC defines the child as every human being below the age of 18 years. The UNCRC provides guidance for the protection of child rights, mostly adopted by cities, nations and the private sector. As a comprehensive body of law relating to child rights, the UNCRC includes, but is not limited to, civil and political rights as well as social, economic, and cultural rights (Akinola, 2019). Consisting of 54 Articles, the UNCRC sets out different ways through which the rights of the child can be protected. Specifically, Articles 3 and 12 detail provisions and inclusion. Contextualising the 12th Article, Lundy (2007) created a model for effective child participation that included space, voice, audience, and influence (Figure 1).

While Lundy's conceptualisation has been widely welcomed in research, policy and practise, there is a dearth of examples in the literature regarding how it can be operationalised (Kennan et al., 2019). For example, in the context of sport, Eliasson (2015) showed that the challenges of child rights abuse is yet to be adequately addressed. There is also evidence that each edition of the Olympic Games in the last decade has seen child rights infringements (Wong, 2011; Caudwell and McGee, 2018). Wong (2011) shows that children are subjected to various forms of abuse in the planning and production stages of MSEs. For example, pre-London 2012, children were in the spotlight when it was discovered that a company in China was using those as young as 12 to make the mascots for the Games. In the sporting realm, violations include child athletes suffering from undue pressure to achieve high performance, resulting in beatings and physical punishment. There is also evidence of sexual harassment and assaults for child athletes (Sanderson and Weathers, 2020). Outside of the sporting context itself, there is also evidence of child labour and trafficking infringements during the planning and delivery of MSEs (Brackenridge et al., 2012). According to Dowse et al. (2018), the invisibility of children within MSE hosting processes suggests that current conceptualisations of the social potential of event hosting are incomplete, representing a significant omission that contributes to the social irresponsibility of many hosting projects. It also raises the question of whose responsibility it is to ensure that the child is adequately represented and provided for in the planning and delivery stages of MSEs. Children are invariably absent from the process of planning and delivering the Games, other than as beneficiaries of the inspirational effects of these sporting spectacles. Brackenridge (2004) argues that the citizenship status of the child is still not fully embedded in all spheres of public life, since their capabilities as decision makers is not universally accepted. For this reason it is still rare to find children consulted or represented in decision-making processes, even in matters of direct concern to them. Instead, parents are consulted, because they play dominant roles in making decisions for their children (Howard and Madrigal, 1990).

And yet, there are some signs of progress that suggest child rights can be built into sport structures and systems, including in the context of MSEs. Partly, this is because of increasing pressure exerted on sport federations to recognise their responsibilities. According to Donnelly (2008), the regulation of child abuse in sport is the reponsibility of both governments and supranational organisations, like the IOC. He argues that since child abuse occurs as an outcome of MSE planning, children should be given precedence in policies. The *Children's Rights in Sports Principles* produced by UNICEF in 2018 provide some clear guidance for event owners and hosts that can strengthen child rights protection standards. These principles suggest that sport organisations, including MSE organisers should: formulate and publish policies committing to the UNCRC protection principles; identify and assess actual or potential adverse impacts on the rights of children and implement measures according to the identified risks; establish specific rules, guidelines, and codes of conduct to implement the policies for respecting and supporting the rights of children set out in Principles 1 to 4, and ensure that they are followed by all persons involved; monitor regularly whether violence, overtraining and other issues that might adversely affect the rights of children occur in the course of sport instruction, practise, and matches; secure reporting mechanisms and remedy channels to address problems (Children's Rights in Sports Principles, 2018).

While these child rights principles have been welcomed (Dowse et al., 2018) and the IOCs awareness of human rights risks has improved in recent years (Grell, 2018), it is necessary to explore in depth the extent to which Olympic Games organisers have enshrined child rights protection measures in their planning and delivery processes. The remainder of this article focuses on the case of the Tokyo 2020 Olympic Games.

## Methodology

Undertaking research on human rights can be challenging because of the sensitivities associated with the subject matter (Powell and Smith, 2009). This study is concerned with the extent to which child rights principles and protocols are effectively embedded in the bidding and planning of the Tokyo 2020 Olympic Games. It is not concerned with investigating the lived experience of children in the host country, because that would require the researcher to be embedded in that context over an extended period of time and have detailed understanding of the language and culture of the children's host environment. To assess the provisions for child rights in the bidding and planning of the Tokyo 2020 Olympic Games, the decision was made to focus on an event being planned during the study period and to subject plans in place to respect, protect and promote child rights to critical scrutiny. The focus on the bid and planning phases for Tokyo 2020 meant that investigations focused on the period between 2011, when the bid process began (the bid was successful in 2013), through to 2020 when the Games were postponed for a year. Though there would be merit in comparing Tokyo 2020 to previous Olympic Games editions, the decision was made to focus on a single case study to enable depth of analysis. Focusing on a single case also enabled the authors to develop greater understanding of the contextual influences on the research topic (Hennink et al., 2020). To fill the research gap and address our guiding research question, we followed an interpretive approach. From an epistemological perspective, interpretivist explanations take a narrative form, allowing the authors to explore the social construct being researched from the perceptions and experiences of participants and stakeholders who have worked closely in that social setting (Thanh and Thanh, 2015). Operationalising that philosophy, the study selected two main research methods to explore the landscape for child rights in planning for the Tokyo 2020 Olympic Games.

First, a systematic documentary analysis was undertaken on child rights-related policies and strategies published in advance of the Tokyo 2020 Olympic Games by the Japanese government, Games organisers and other relevant stakeholders. These documents, detailed in Table 1, were accessed and downloaded online. Second, seven semi-structured interviews were conducted with strategic actors with an expertise or interest in child rights in relation to the Tokyo 2020 Games. Interviewees included senior management representatives from five NGOs (all focussed on child rights protection in the areas of child labour, trafficking and child athlete protection), one member of the Games Organising Committee, and an independent child rights lawyer who also represents a human rights coalition organisation. All NGOs operate on an international level, and have offices in Japan. The number of interviewees can be considered as adequate because of their knowledge of the topic being researched (Müller, 2015). Due to the restrictions on travel caused by the coronavirus pandemic interviews were conducted using remote video calling platforms, with each lasting between 45 and 60 min. Interviews were conducted with a focus on themes arising from the documentary analysis alongside insights from Lundy's (2007) model. Interviews adopted a conversational style for the purpose of flexibility and depth (Qu and Dumay, 2011; Raworth et al., 2012). Questions centred on the themes of provision, protection and child participation in the decision-making processes of the Tokyo 2020 Olympic Games. Depending on their area of expertise, interviewees were asked slightly different questions. For example, those with legal expertise were asked to comment on the effectiveness of planned measures to protect child rights. Those advocating for child rights from NGOs and CSOs were asked to comment on the extent to which Games planning (from government and the Organising Committee) adhered to international standards and whether they were consulted in the process. Finally, organisers were asked to detail what child rights procedures and protocols were part of their planning horizons and what challenges were faced in embedding child rights in the Tokyo 2020 Olympic Games.

**Table 1:** List of documents reviewed.

**Document name**	**Author**	**Year of publication**
**Human rights documents**
Sporting Chance White Paper	Terre Des Hommes and UNICEF	2017
Child Rights in Sports Principles	Japan Committee for UNICEF	2018
Human Rights Watch Report	Human Rights Watch	2020
**Olympic bid documents**
Candidature Acceptance Procedure for Host Cities	IOC	2011
Reports of the IOC evaluation commission	IOC	2013
Tokyo 2020 Joinder Agreement	TOC	2013
Tokyo 2020 Sustainability Plan Versions 1 and 2	TOC	2017
Host City Contract–Operational requirement	IOC	2017
Host City Operational requirement	IOC	2018
Japan human rights commitments and pledges	Japanese Government	2019
Japan 2020 Olympic Guidebook	TOC	2019
The Olympic Charter 2019	IOC	2019
**Host city contracts**
Host City Contract	IOC and JOC	2013
Appendix 1 and Addendum to the Host City Contract	IOC and JOC	2017
Addendum No 2. To the Host City Contract	IOC and JOC	2017
Addendum No 3. To the Host City Contract	IOC and JOC	2019

Analysis of documents and interview transcripts was undertaken using the qualitative software tool, Nvivo 12, 64–bit version. Initially, documents and transcripts were read multiple times by the lead author to ensure full immersion in, and understanding of, the data, an important element of qualitative research (Green et al., 2007). After multiple readings, the transcripts and documents were analysed thematically (Braun et al., 2016), using Nvivo 12, 64–bit version to create theme nodes (Hilal and Alabri, 2013). An open and inclusive coding approach was adopted (Smith and Firth, 2011), identifying and labelling all segments of interest and relevance within the dataset, and everything of relevance within those segments (Terry et al., 2017). Co-authors were then involved in an independent checking process to confirm the appropriateness of the selected themes.

Ethically, the research was considered low risk by the University's Ethics Committee, primarily because no children were involved as interviewees. In conducting the study, all ethical principles that apply to research were adhered to. Sensitivities were identified around participant confidentiality and these were addressed through the use of identifiers (e.g., Interviewee A) in place of participant's real names and organisational affiliations (see Table 2).

**Table 2:** Interview list.

**Identifier**	**Role**
Interviewee A	Representative of the Tokyo 2020 Organising Committee
Interviewee B	Child rights lead at an International Human Rights Organisation
Interviewee C	Child Rights Advocate and athlete protection lead
Interviewee D	Child Rights Lawyer/coalition member of an international human rights organisation
Interviewee E	Policy developer for a Child Rights Charity Organisation
Interviewee F	Child labour chair for an international Child Rights Organisation
Interviewee G	Child trafficking representative for an international Child Rights Organisation

## Findings and Discussion

The analysis of documents and interviews generated several themes relating to enabling child participation in decision-making processes and embedding child rights protection in policies. First, in this section, the theme of participation will be discussed before attention turns to the theme of provision for protection. These themes are consistent with those identified in the UNCRC and Lundy's (2007) conceptual model of participation–both of which highlight the guidelines and principles for the rights of children in all sectors, including MSEs.

### Child Participation and the Tokyo 2020 Olympic Games

In Figure 1, Lundy (2007) set out the requirements for meaningful child participation, which included the right to express a view (space and voice) alongside the right to have views given due weight (audience and influence). Findings from our study revealed little evidence of child participation considerations in the official policies or strategies for the Tokyo 2020 Games, or in the views of key stakeholders. Lundy's (2007) model of participation was developed to help practitioners meaningfully and effectively implement a child's right to participate by focussing on the distinct but interrelated elements of Article 12 of the UNCRC (Kennan et al., 2019). Involving children in the decision-making processes is an integral part of child participation (Lundy, 2007). However, exploring the policy environment for Tokyo 2020, the evidence suggests that participation was mainly concerned with encouraging young people to participate in sport, rather than ensuring children have a voice or an audience in planning and decision making. This is illustrated in the Candidature File, otherwise known as the Tokyo bid book, which noted that by hosting the Games, Tokyo 2020, “will do its best to ensure that both old and young people will be encouraged to participate not only in the 2020 Olympic Games, but also enjoy the sporting events” (Candidature File, Vol 1, p. 006). Also in the Candidature File, the Tokyo 2020 bid team identified a series of initiatives to encourage children to participate in the Games, mainly as spectators through socially–geared education programmes. These initiatives targeted children from areas impacted by the Great East Japan Earthquake, and those attending special needs schools, funded by the Tokyo Metropolitan Government (Candidature File, p98). Commitment to participation-by-spectating was a feature of the bid:

One of Tokyo 2020's objectives is to promote Olympism to young people by encouraging them to participate in the Olympic and Paralympic celebrations and Tokyo 2020 will adopt successful policies such as London 2012's lower charge for children (Candidature File, Vol 1, p. 100)

This commitment was corroborated by Interviewee A, a member of the Tokyo 2020 Organising Committee who stated that:

One of our visions is to encourage children to participate as much as they can. I can confirm to you that Tokyo 2020 has child volunteers and participants already

At the bid stage, alongside these participation-focused initiatives, Japanese organisers also proposed strategies to make tickets cheaper for children, increasing the accessibility of the Games. While initiatives to encourage child participation, physically, in the Tokyo 2020 Games are commendable, for participation to be meaningful it needs to transcend physical participation (Lundy, 2007). Young people are often heralded as the main beneficiaries of the inspirational effects of the Olympic Games. However, given the growing evidence of child rights infringements related to the planning and delivery of MSEs, Dowse et al. (2018) suggest that more needs to be done to secure the active involvement of children in the planning phase if Lundy's (2007) ambition for space, voice, audience and influence is to be realised.

A prerequisite for the meaningful engagement of children and young people in decision making is the creation of an opportunity for involvement; a space in which children are encouraged to express their views (Lundy, 2007). As Dolev-Cohen et al. (2020) note, many abused children are left bearing the brunt of their abuse alone, because there is no place where they can feel safe to report and divulge information about their experiences. It is common practise to refer to parents in discussion of child rights, involvement in decision making and reporting abuses, especially since parents play dominant roles in shaping the participation decisions of their children (Howard and Madrigal, 1990). In the context of Tokyo 2020, there were no communication and reporting mechanisms for abuse in place prior to 2020. At that point, under pressure from a negative Human Rights Watch report, reporting hotlines were set up by the Japanese government. However, interviewees cast doubt on the effective functioning of these reporting mechanisms. As Interviewee D, a child rights advocate stated when asked about the child abuse reporting mechanisms available in Tokyo in advance of the Olympic Games, “The hotlines are mainly not accessible and only available at limited times [between 1 p.m. and 5 p.m.], the reporting mechanism created is not friendly for child athletes.” There are two points worth making here. First, there was no evidence of proactivity on behalf of organisers to provide a space for voice and influence in planning. The reporting hotlines were a reactive response to external pressure. Second, when the reporting mechanism was introduced it did not adequately engage with those likely to make use of it (i.e., children) and as a result it proved to be ineffective.

We found only limited evidence that Games organisers and the government were serious about the importance of strengthening child rights protocols, reacting late in the planning process. Interviewee A, a member of the Tokyo 2020 Organising Committee, suggested that:

The Japanese government is intentional about child rights provision, which is why the government and the sports community embraced the Children Rights in sports Principle when it was introduced. The Japanese government has also always embraced NGOs who have come forward with their suggestions as regards the Tokyo 2020/21 Games

In addition, Interviewee A also noted that the Japanese government was working on creating a safe space for children, especially child athletes, where children would be encouraged to speak out and share their opinions but confirmed that this safe sport centre had not been established by 2020 when the Olympic Games were due to take place:

Based on the current report by Human Rights Watch, the government has gotten curious about doing something in the new legislature about the child/child athlete. One of the ideas we came up with is to establish a child safe sport centre for child athletes during the Olympic Games, and then we can enhance the idea to include purposes for it to become a legacy for child athletes in a broader way (Interviewee A)

Another element of Lundy's (2007) child participation model that was missing from the Tokyo 2020 Olympic Games planning process was “voice.” As stated in the UNCRC, Article 12, children should be assured of the opportunity to express their views freely (Lundy, 2007). As suggested by Interviewee D, a child rights advocate in Japan, “some of the most voiced concerns of child participants in the Olympic Games, especially child athletes, is that they might lose their spot if words get out that they had spoken to authorities.” This, they went on to suggest, was due to the importance of preserving a culture of harmony in Japanese society:

When a child feels like they have been exploited, they will not speak out because they will stand out. Someone will make a fuss and they will embarrass their family in that way. Even if the family will not feel embarrassed, the perception of the child is that they have brought embarrassment to their family. Harmony is really important in the Japanese culture, and they will do anything to preserve that (Interviewee D)

Five out of the seven interviewees mentioned that younger generations in Japan are usually not keen to voice their opinions, even to child rights organisations. For example, Interviewee F, a child rights advocate and campaign manager of an international human rights organisation, highlighted the difficulties their organisation had faced when attempting to provide a platform to voice concerns and contribute to their protection:

Approaching children in Japan was difficult; hence, we were not able to get any child to contribute to our child protection campaign. They are either with their parents or being chaperoned by school guardians

Interviewee D put the gap between rhetoric and reality of voice down to the hierarchical structure of Japanese culture. He suggested that there are just a few people who can articulate their opinions or speak up to authority figures:

Especially the younger generation in Japan, they can hardly speak to their management or senior people due to the hierarchy (Interviewee D)

More importantly, none of the Tokyo 2020 Olympic Games strategic documents made reference to child stakeholders, child athletes, or child athletes' representatives being consulted in the formation of policies or their implementation. Interviewees suggested that considering the hierarchical nature of Japan life, having representatives speak on behalf of children, would have been a more effective approach for the Tokyo 2020 Games organisers to take:

The only way to get their opinion would have been by legal representation, where they know that they are protected (Interviewee D)

However, other child rights representatives suggested that child athletes would think twice before speaking to legal representatives because “they will be scared of losing their spot in the Olympics if word gets out that they have reported their coaches or spoken to someone about how they're being treated” (Interviewee C, athlete protection lead). This speaks to the importance of creating a safe space for children to have a voice and access to safe representation in decision-making processes. Children will often need the help of others in order to form a view (Lundy, 2007). While Everley (2020) suggests that representation of the vulnerable by interested adults is an important factor in supporting safe engagement, she also argues that this potentially means that those responsible for children may prioritise success above welfare.

While the general commitment to children's participation in the Tokyo 2020 Games, as spectators, volunteers and recipients of educational programmes is to be welcomed, analysis of bidding and planning documentation found little formal commitment to respecting, protecting or promoting child participants' rights.

### Child Rights Protection and Tokyo 2020 Olympic Games

The UNCRC sets out very specific expectations relating to protection of the child. Article 19, in particular, speaks to the importance of protecting children from violence, abuse and neglect. Furthermore, UNICEF's *Children's Rights in Sports Principles* also provide guidance on how sport organisations can better protect children. MSEs are known to be one of the places where abuses occur against children, hence the need for event owners and organisers (including host governments) to proactively plan in child rights protection measures when hosting an event on the scale of the Olympic Games.

Our study findings indicate that Tokyo 2020 Olympic Games strategies and policies contained little evidence of protection strategies directly related to children–either in sport or to address the wider harms from organising. Moreover, those measures that were put in place only emerged in the latter stage of event planning. Policies that have contractual power, including the Host City Contract (HCC) and Candidature Acceptance Procedure contained almost no mention of specific child rights measures put in place for the Tokyo 2020 Olympic Games. Both contractual documents mentioned general measures that applied to children but these were not primarily directed at protecting child rights, per se. This can also be linked to the lack of child rights specificity in the Olympic Charter and other strategic documents governing the selection of host cities and the formation of the HCC. When asked to comment on the lack of specific child rights protection measures in the Tokyo 2020 policies and guiding documents, Interviewee B, child rights lead at an international human rights organisation, specifically mentioned the importance of child rights representation in guiding policies. In their words, “embedding specifics of child rights protection in policies would be a move in the right direction.” Similarly, Interviewee C noted that children are often neglected, and it was time for event owners and organisers to be intentional about child rights in their policies, starting with the IOC. This was corroborated by Interviewee B who noted the importance of getting children adequately protected through policies:

In terms of child rights, it is really important that it's integrated with human rights. It has to move together otherwise it will not hold water. Even though children have an appeal, and people tend to include them faster in events, protecting them also needs to be done with care. This is why those policies are important, they are written guidelines that can be referred to by organisers and events planners (Interviewee B)

When rights protections were mentioned in the HCC, these were primarily about commercial rights and the role of the host city in protecting the IOC and its partners by passing exceptional legislation around ambush marketing and related activities. Recently, after the IOC strengthened its human rights requirements for the Paris 2024 Olympic Games edition, Tokyo 2020 organisers were asked to respond. Though not contractually obliged, organisers introduced limited additional commitments to respecting human rights, labour and business practises in the Tokyo 2020 Sustainability Plan, Version 2. Under the “consideration of human rights” section of this Plan, this increased focus on human rights focus was associated with the IOCs adoption of Olympic Agenda 2020:

The Olympic Agenda 2020 also states that the host city contract should include clauses with regard to the Fundamental Principle 6 of the Olympic Charter as well as to environmental and labour-related matters and Host City contracts after Paris 2024 includes compliance with the UN Guiding Principles on Business and Human Rights (The Tokyo 2020 Sustainable Plan Version 2, p. 74).

Additionally, the Sustainable Plan Version 2 document noted that Tokyo 2020 aimed to avoid causing or contributing directly and indirectly to discrimination and human rights abuses through the entire Games-related process. As a form of provision to prevent these infringements from happening at the Games, the Sustainable Plan Version 2 proposes that organisers would “prepare a communication system and properly understand the situation of human rights consideration issues” (p. 75) and in the event of infringements occurring it would “proactively request correction to abusers and protect victims” (p. 76). However, there were no details as to how such remedies would work in practise, a weakness that often accompanies broad human rights commitments (MSE Platform, 2018).

While the amendment to the Sustainable Plan suggests that human rights provisions were strengthened in the latter stages of planning for the Tokyo 2020 Olympic Games, interviewees challenged this progressive rhetoric, highlighting the inadequacy of reporting systems and remedy measures to identify and address child rights infringements that might arise. First, a child rights organisation in Japan, when asked about the protection mechanisms in Japan, and how efficient they were, noted that the reporting hotlines set up to report child rights abuses were only available in Japanese. This suggests that these hotline provisions can only serve Japanese athletes. In their words:

When we attempted to call these lines, the only available language was Japanese. This means that child athletes, possibly from other nations, who do not understand the Japanese language cannot report (Interviewee C)

We realised that some of these calls are not free like they were campaigned to be. We had to pay to speak to someone on the hotline. How are minors expected to get the fund to be able to report? (Interviewee F)

Given the Olympic Games is a global event, this limited the availability and usefulness of the hotline to athletes coming from outside of Japan. Second, in its 2020 report, titled “I was hit so many times I can't count,” Human Rights Watch also noted that the hotlines were only available between 2 p.m. and 5.p.m. on Tuesdays and Thursdays, significantly restricting access. Third, an independent child rights expert in Japan also noted that:

After all these years, we have no statistics about the usage of the hotline (Interviewee D)

These findings indicate that while the IOC appears to have strengthened its human rights commitments, now requiring host cities to adhere to the UN Guiding Principles on Business and Human Rights, this does not automatically lead to improvements at the host city level. Unless there are contractual obligations for host governments and organisers relating to human rights then the IOCs leverage is diluted (McGillivray et al., 2021). Agenda 2020 reforms and pressure from independent NGOs do not automatically put children at the centre of rights protection in the planning for MSEs. Interviewee B suggested that embedding child rights protection measures in policies must be an intentional act. Even if NGOs have to step in, there must be a guiding policy in place before delivery of MSEs. Moreover, policy rhetoric around child rights provisions does not automatically translate into action (Interviewee B). There is a need for political will and leadership to ensure that policy provisions are carried into practise (Interviewee C).

Depending on the prevailing human rights culture in a host country, child rights might be higher or lower on the agenda. Interviewees suggested that in Japan, recognition of child rights remains quite low on the political and social agenda, particularly in relation to sport. When this is case, according to Hong (2004):

In a society where the parents, the coaches and teachers, and above all, the party and the government, believe that they all have absolute authority over children and where the progress of the children (in this context, participating in the Olympics) is essential to the greater good, children's rights will hardly become a priority (pg. 350).

In the case of Tokyo 2020, the HCC did not include specific human rights or child rights clauses, meaning that it was left largely to the organisers to decide how best to address potential infringements. Our findings suggest that organisers had not put in place robust measures to identify rights infringements, or to implement reporting procedures to address potential child rights concerns should they arise. This reinforces Dowse et al.'s 2018 view that too often it falls on the host cities to ensure that human rights protection measures are adequately provided before the Olympic Games. In the case where the host cities have not done enough to ensure child rights protection, NGOs have been known to collaborate with both organising committees and host cities (McGillivray et al., 2019) to help strengthen child rights protection measures. Our study found evidence that this has also been the case with the Tokyo 2020 Olympic Games. For example, after realising that the hotline communication measure in Tokyo 2020 had its shortcomings, advocacy organisations had to step in to resolve the issue. For example, Interviewee E provided details of the alternative hotline they had to put in place:

Our hotline is entirely free. We have been working with our current partners in Tokyo. Although we do not anticipate a high volume of child abuse in the Games, because of the pandemic situation. However, regardless, we want to be ready, we want to have something in place for the children who will be involved in the Games. Moreover, this is not exclusive to child athletes and children in the host communities; we are also concerned about child athletes and visitors who will be attending the Games from other parts of the world

Furthermore, Human Rights Watch, in an independent report, making reference to the limitations of the hotline and cases of abuse of Japanese child athletes, called on the Japanese government and Japanese sport organisations to implement standards to prevent child athletes abuse before the Tokyo 2020 Games.

This evidence of reactive responses and workarounds to address institutional failings reinforces McGillivray et al.'s 2019 claim that responsibility for avoiding rights infringements at MSEs often extends to NGOs, CSOs, and other actors in addition to Games organisers. This is primarily because of the historical lack of strategic leadership from MSE awarding bodies and games organisers to foreground human rights.

## Conclusion

This study's findings highlight a gap in the way children are considered, particularly in the bidding and planning stages leading up to hosting the Olympic Games. Our study found some limited evidence of general human rights protective measures being embedded in the Tokyo 2020 Olympic Games planning documents and functional areas, especially in the months before the Games were due to take place. However, there was a dearth of specific child rights protection initiatives and those that were established seemed to represent an afterthought, established in response to external pressure. As Donnelly (2008) has suggested, children need to be treated with much more importance, and given precedence in policies guiding MSEs if their rights are to be respected, protected and promoted. It is important to acknowledge that significant progress has been made in how children are protected in sport, with many agencies now taking an active role in prevention work (Brackenridge and Rhind, 2014). However, these agencies differ in their focus and it is important to acknowledge that rights issues occur in and outside of sport. Agencies can be characterised broadly as sport-specific (focussing on abuse prevention in sport), children's rights organisations (focussing on child protection around sport events) and humanitarian organisations (focusing on child development and protection through sport (Brackenridge and Rhind, 2014). Our study suggests that MSE organising committees are often left to their own devices, expected to adhere to broad human rights principles without the necessary expert support to develop robust and sustainable policies and protocols that are amenable to monitoring and the development of remedy measures. As a result, NGOs and CSOs are forced to enter the fold at a stage when rights infringements are already materialising to shame and blame organisers and pressure them into strengthening their rights protections (McGillivray et al., 2021).

Relying on a reactive model for human rights protections risks child rights being infringed before and during MSEs. Leveraging the power and resources of MSEs early and building measures and remedies into the event planning process is necessary if host cities are to be held to account more effectively (Heerdt, 2018; McGillivray et al., 2021). Ultimately, if strengthened human rights requirements can be written into the IOC's HCC then there is a greater likelihood that these events will take rights more seriously as they are planned and delivered (Kassens-Noor and Lauermann, 2017). Binding guarantees are required to close the accountability gap and improve access to remedies for human rights abuses associated with MSEs (Heerdt, 2018).

While our study suggests that Tokyo 2020 organisers were largely reactive to child rights issues arising in relation to the event, there are some positive signs of progress in the MSE human rights landscape, more generally. In recent years the IOC has been subject to intensive media pressure to follow FIFA in requiring bidders to include detailed human rights risk assessments in their submissions (Bason and Grix, 2018). These risk assessments require potential hosts to consider a range of rights issues, including consideration of the rights of children in the host country as the Games are planned and delivered. There are also signs that awarding bodies and organisers now recognise the need to consider child rights in the sporting field and when thinking about the impact of planning the Games on the host country or city itself (e.g., infrastructure, education and the visitor economy). Crucially, developments like the *Children's Rights in Sports Principles* have strengthened the case for regular independent monitoring processes at agreed intervals during MSE planning processes and emphasised the importance of remedy for those whose rights have been infringed.

In the final stages of planning for the Tokyo 2020 Olympic Games we found that Games organisers, politicians and those in positions of power were aware of growing media interest in host organisers personal conduct and the experience of the Games for citizens, athletes and the international media. The organisation of the delayed Games was shrouded in controversy, with high profile resignations over personal conduct that compromised commitments to equality, diversity and inclusion. Because Tokyo 2020 was awarded to Japan before the IOC introduced its human rights policy (in 2017) and strengthened its protocols to respect, protect and promote human rights as part of the bid and delivery process, these Games appear to have paid lip service to the issue of child rights. Analysis of formal policies demonstrates a misalignment between generalised commitments to human rights and child rights conventions and (a lack of) specific plans in place to govern the 2020 Tokyo Olympic Games. When the IOC eventually sought to influence the Japanese government and Games organisers to strengthen its human rights measures in the 2 years leading up to Tokyo 2020 they were ineffective because they were unable to hold them contractually accountable.

Available evidence suggests that human rights need to be enshrined in the contractual obligations of host cities or nations from the bid stage onwards if they are to be leveraged effectively (Heerdt, 2018; McGillivray et al., 2021). The same applies to child rights. If Games organisers are required to consider the potential risks to children in sport and as a result of planning the Games, then there is a greater chance of child rights being part of planning, including having children involved in decision making processes.

To turn that rhetoric into reality, a number of practical measures could be introduced. First, MSE bid committees should create reference groups, akin to a children's panel, to inform the bid process, providing space and voice (Lundy, 2007) at an early stage of the Olympic cycle. Second, this reference group should then find a place in the governance structures of the organising committee to enable continuity, ensuring child rights issues remain high on the agenda during the planning and delivery phases. Third, the reference group should have representation from international, national and local child rights specialists so that learning from previous MSE experiences can be integrated with local contexts. Fourth, the reference group needs to have participation from children and not simply reinforce adult-centric views and opinions. Finally, it is important that reference groups like these have some influence and that there is accountability built into governance processes so that concerns can be surfaced and acted upon in an open and transparent manner.

Before closing, it is important to recognise study limitations and to outline areas for further research. The main limitation of this study lies in the number of interviews conducted. The initial plan was to go on a one–month observation visit to Tokyo, Japan. However, this plan was interrupted due to the impact of the coronavirus pandemic. This limited the research, as the lead researcher was unable to gather data in the host location. However, the lead researcher was able to work around the limitation by getting in contact with specific stakeholders and organisations currently based in Japan, either through referrals or by searching on official organisational websites.

Future research into the child rights agenda at MSEs should consider a more embedded, participatory approach to provide a more child-centric perspective. This will require the development of relationships, over time, with child-focused organisations to build trust and the use of methodological tools that reflect the preferences of the children being targeted. Further research could also fruitfully consider comparative analyses, drawing on past and future Olympic editions so that continuities and discontinuities can be more effectively contextualised.

## Data Availability Statement

The original contributions presented in the study are included in the article/supplementary material, further inquiries can be directed to the corresponding author.

## Author Contributions

OA and DM contributed to the conceptions and design of the study. OA carried out the research and wrote the first draft of the manuscript. DM, GM, and SC supervised the research. All authors contributed to manuscript revision, read and approved the submitted version.

## Conflict of Interest

The authors declare that the research was conducted in the absence of any commercial or financial relationships that could be construed as a potential conflict of interest.

## Publisher's Note

All claims expressed in this article are solely those of the authors and do not necessarily represent those of their affiliated organizations, or those of the publisher, the editors and the reviewers. Any product that may be evaluated in this article, or claim that may be made by its manufacturer, is not guaranteed or endorsed by the publisher.

## Abbreviations

IOC: International Olympic Committee
UNICEF: United Nations Children's Fund
CSO: Civil Society Organisations
NGO: Non-Governmental Organisations
UDHR: Universal Declaration of Human Rights
UNCRC: United Nations Convention on the Rights of the Child
SRA: Sport and Rights Alliance
NOC: National Organising Committee
OCOG: Organising Committee for the Olympic Games
TOCOG: Tokyo 2020 Organising Committee for the Olympic Games
HCC: Host City Contract
OC: Olympic Charter.
